# Effects of Migration Distance on Shifting Migratory and Breeding Phenology in Waders

**DOI:** 10.1002/ece3.73080

**Published:** 2026-02-27

**Authors:** Verónica Méndez, José A. Alves, Jennifer A. Gill, Böðvar Þórisson, Camilo Carneiro, Aldís E. Pálsdóttir, Sölvi R. Vignisson, Gunnar Tómasson, Tómas G. Gunnarsson

**Affiliations:** ^1^ South Iceland Research Centre University of Iceland Laugarvatn Iceland; ^2^ Department of Biology & CESAM—Centre for Environmental and Marine Studies University of Aveiro Aveiro Portugal; ^3^ School of Biological Sciences University of East Anglia, Norwich Research Park Norwich UK; ^4^ Natural Science Institute of Iceland Garðabær Iceland; ^5^ Independent Scholar Selfoss Iceland

**Keywords:** arrival time, arrival‐laying gap, pre‐breeding period, shorebirds, spring temperature, timing of breeding

## Abstract

Shifts in phenology are widely reported across taxa and, among migratory birds, advancing timing of breeding has occurred predominantly in short‐distance migrants. Long‐distance migrants might be less able to advance breeding if they arrive later and breed soon after arrival, but opportunities to quantify trends in phenology across species that experience similar breeding conditions but vary in migration distances are rare. Between 2007 and 2022, we recorded arrival and laying dates across lowland Iceland for nine wader species that vary in migration distances. Waders wintering closer to Iceland arrived ~6 weeks earlier than those wintering further away, yet laying dates differed by only ~1–2 weeks. Over this survey period, short‐distance migrants advanced laying despite little or no advance in arrival, while long‐distance species advanced both arrival and laying dates. The longer arrival‐laying interval in species travelling shorter distances appears to allow earlier laying in warm springs, a flexibility less available to later‐arriving species. Due to the benefits of breeding early in migratory systems, the opportunity of early nesting in warming springs could be contributing to divergent population trajectories of short‐ and long‐distance migrants. Quantifying the phenology of nest and fledging success of species migrating over different distances will help to identify the costs of travelling further and arriving later during this period of rapid environmental change.

## Graphical abstract

Short‐distance migrants arrive earlier and are advancing laying at a faster rate than long‐distance species, particularly in warmer springs. Early arrival does not always result in earlier laying, but short‐distance migrants can exploit favourable conditions and lay before many long‐distance migrants arrive. Migration‐timing constraints may therefore limit access to early breeding opportunities and contribute disproportionately to population declines in long‐distance migrants.

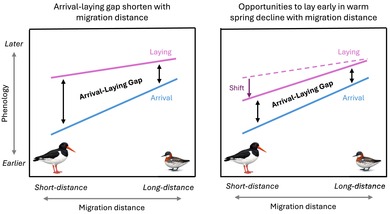

## 1 Introduction

Rapid climatic and environmental changes across the globe in recent decades have led to a suite of responses, including changes in phenology, distribution, and abundance, that vary among species (Knudsen et al. 2011; Ovaskainen et al. 2013; Parmesan and Yohe 2003; Walther et al. 2002). Among migratory birds, shifts in the timing of spring migration and timing of breeding are widely reported (Both et al. 2010; Gill et al. 2014; Gunnarsson and Tómasson 2011; Morrison et al. 2015), but the magnitude of these shifts varies greatly among species. This variation is frequently associated with migration distance, with species travelling further often showing little or no phenological change (Miller‐Rushing et al. 2008; Rubolini et al. 2007). Understanding why phenological change tends to be weaker among longer‐distance migrants is particularly important, as many of these species are in decline (Franks et al. 2018; Moller et al. 2008; Vickery et al. 2023; Zurell et al. 2018). Limited shifts in phenology, and the associated demographic consequences, may be contributing to these declines.

Avian species breeding at northern latitudes are constrained by the short time period available to incubate eggs and raise young, particularly migratory species that also need to prepare for and complete post‐breeding journeys. However, within this relatively short breeding season there can be substantial variation among species in both the timing of spring arrival into the breeding areas (Gunnarsson and Tómasson 2011) and subsequent laying dates (Gill et al. 2014; Saalfeld and Lanctot 2017). This variation is also likely to be related to differences in migration distance, as species that undertake longer journeys tend to arrive and breed later than those that winter closer to their breeding areas (Both et al. 2006; Gill et al. 2014; Gunnarsson and Tómasson 2011). Moreover, whereas shifts in the timing of arrival of long‐distance migrants appear to be less associated with temperature changes at their breeding destinations (Both et al. 2010; Gill et al. 2014; Gunnarsson and Tómasson 2011; Zaifman et al. 2017), the timing of laying in both long‐ and short‐distance migrants is often associated with the onset of the spring growing season (Tavera et al. 2024). This suggests that both groups respond to local conditions with regard to laying phenology, but the extent of these adjustments under the same environmental conditions remain unexplored.

The period between arrival on the breeding grounds and laying may play an important role in facilitating or constraining migratory species' responses to climate change, as early arrival can potentially provide opportunities to breed early should conditions allow, while the shorter time gaps between arrival and laying that long‐distance migrants typically experience may limit their capacity to advance laying dates (Gill et al. 2014; Low et al. 2019). Given the potential advantages of early breeding (including increased time available for replacement clutches following nest loss, higher breeding success and increased rates of offspring recruitment; Alves et al. 2019; Carneiro et al. 2021; Morrison et al. 2019), differences in the capacity to advance breeding could have important consequences for demography and population trends (Gilroy et al. 2016; Vickery et al. 2014). However, arrival and laying phenology are often studied separately and within populations (Carneiro et al. 2023; Lourenço et al. 2011; Low et al. 2019; Nightingale et al. 2024; Smith and Fraser 2024), limiting our capacity to understand the links between these two key events and how they vary across species and migratory strategies.

The Icelandic wader community provides a good opportunity for examining relationships between migration distance, timing of spring arrival, timing of laying, and local weather conditions, as it includes species that migrate to locations up to 10,000 km as well as others that remain in or near Iceland and breed sympatrically across the lowland areas. A very large proportion of the global populations of several of these wader species breed in Iceland (e.g., 45%–61% of Golden Plover ( *Pluvialis apricaria* ) and 19%–28% of Whimbrel ( *Numenius phaeopus* ); Skarphéðinsson et al. 2016), and given that many of these populations are currently declining (Pálsdóttir et al. 2025), understanding their responses to changing conditions is therefore of major conservation importance.

Using long‐term datasets (2007–2022) of migratory and breeding phenologies for nine Icelandic breeding waders, here we explore whether species that migrate over shorter distances (1) arrive and lay earlier, (2) are advancing timing of arrival and laying more, and (3) have stronger associations between local spring temperatures and timing of arrival and laying than species that migrate further.

## 2 Materials and Methods

### 2.1 Estimating Migration Distance

**FIGURE 1:**
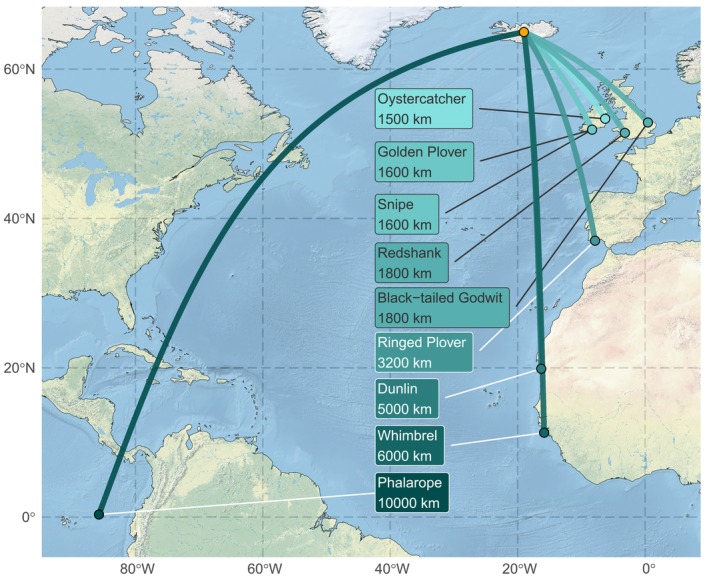
Migration distances of nine common wader species breeding in Iceland, considered in this study. Migration distance is measured as the distance (great circle route) between the approximate mid‐point in Iceland (orange point) and the representative wintering location holding the highest concentration of the Icelandic population. The representative wintering location used for each species is shown in green, with lighter colours representing shorter migration distances and darker colours representing longer distances.

The datasets used in this study contain phenological information for nine common wader species that breed across the Icelandic lowlands and migrate over different distances. For each species, migration distance was considered as the distance between the approximate mid‐point of Iceland and the wintering area that holds the highest concentration of the Icelandic population (termed representative wintering location), estimated based on ringing recoveries, tracking studies, and population‐level distribution summaries (see references below for each species), and was calculated as the great circle distance using R package *geosphere* (Hijmans 2021). The wintering ranges and representative wintering locations used for distance calculations were as follows: Eurasian Oystercatcher (hereafter Oystercatcher): range = Iceland to N Spain (representative wintering location = W Ireland) (Gunnarsson and Tómasson 2011); Snipe ( *Gallinago gallinago* ): Britain and Ireland (S Ireland) (Franks et al. 2022; Gunnarsson and Tómasson 2011); Golden Plover: Britain and Ireland to Iberian Peninsula (S Ireland) (Gudmundsson 1997; Gunnarsson and Tómasson 2011); Redshank ( *Tringa totanus* ): Britain and Ireland to N Spain (S Wales) (Franks et al. 2022; Gunnarsson and Tómasson 2011); Black‐tailed Godwit: Britain and Ireland to Iberian Peninsula (E England) (Alves et al. 2013a; Gill et al. 2002; Gunnarsson and Tómasson 2011); Ringed Plover: NW France to W Africa (S Portugal) (Franks et al. 2022; Thorisson et al. 2012); Dunlin ( *Calidris alpina* ): NW & W Africa (W Mauritania) (Franks et al. 2022; Pienkowski and Dick 1975); Whimbrel: Iberian Peninsula to Sierra Leone (W Guinea‐Bissau) (Alves et al. 2016; Carneiro et al. 2021; Gunnarsson and Tómasson 2011); and Red‐necked Phalarope: west coast of central America to northern Humboldt Current (off west coast of central America) (van Bemmelen et al. 2019) (Figure 1). Because wintering distributions vary in spatial extent among species and individuals, these locations represent population‐level central tendencies rather than exact wintering sites for all individuals, but provide a robust proxy for relative migration distances among species.

### 2.2 Determining Species First Arrival Dates Into Breeding Areas

In southern Iceland, first arrival dates (FAD) have been recorded at Laugarás (64° 7′ N, 20° 30′ W) for most of the species, and Laugardalur (64° 13′ N, 20° 43′ W) for Ringed Plover, Dunlin, and Phalarope, since 1988 and 2018, respectively. Each day from early March until the end of May, a standardised observation route was walked and driven four times a day throughout the arrival period of these species at both sites and first individuals of each species seen or heard were recorded (more detail in Gunnarsson and Tómasson 2011). To match the arrival dataset with the laying dataset, we only used FAD data from 2007 to 2022 (see below).

As these locations were inland (~40 km from the coast), the data are unlikely to be confounded by the occasional very early arrivals that occur along the coast each year and should thus be representative of the main wave of spring migration in Iceland (Gunnarsson and Tómasson 2011). For example, in Black‐tailed Godwits, a species whose full arrival period has been monitored in detail since 1999, the first arrivals at this location closely match the modal values of the total arrival curve (Gunnarsson et al. 2006) and correlate with records of the first colour‐ringed individuals on arrival locations in coastal Iceland (Gill et al. 2014), suggesting that this survey captures annual variation in arrival patterns well.

### 2.3 Estimating Egg Laying Dates

From April each year (2007–2022), nests of all the species were located through surveys across lowland Iceland throughout the breeding season. Laying date (i.e., the date when the first egg was laid) was estimated by back‐calculating from incubation stage, using standard egg‐floating techniques (Liebezeit et al. 2007) and assuming species‐specific incubation period and egg laying interval (Table S1); or observed hatching dates (14% of monitored nests that survived until hatching). GPS positions were recorded for most nests (*n* = 5859), but for 134 nests, only the region where they were found was documented (S, SW, W, NW, NE; Table S2).

The potential influence of renesting events on lay date estimates was reduced by removing all known second and later breeding attempts of pairs that were identified with an earlier nest in the same season (Table S1), for individuals marked with unique combinations of colour‐rings (Alves et al. 2012; Carneiro et al. 2019; Méndez et al. 2020; Thorisson et al. 2012). As renests tend to occur in the later part of the season (Saalfeld and Lanctot 2017), we also explored the effects of including ‘late’ laying dates for each species and year (those that were > 1.5 times beyond the interquartile range of laying dates above the 75th percentile; Messmer et al. 2021). Exclusion of the resulting ‘late’ nests did not alter the outcome of the analyses of laying dates (Table S3); thus, the larger dataset is presented here.

### 2.4 Spring Temperature Data

To explore the influence of spring temperature on migratory and breeding phenology, we extracted the monthly mean of the daily mean temperatures (in ^o^C) from March to June for the period 2007 and 2022 from five weather stations operated by the Icelandic Meteorological Office (www.vedur.is): Eyrarbakki (S), Keflavik (SW), Reykjavik (W), Bolungarvik (NW), and Raufarhöfn (NE); the closest stations to the breeding study locations. We then used the mean of the March and April mean temperatures to estimate the temperature during arrival (arrival temperature, at), and the mean of April, May, and June to estimate the temperature during laying (laying temperature, lt), periods that reflect the arrival and nesting periods of the focal species in Iceland. We used temperature as a surrogate for spring conditions as it influences the condition of inland waters and soils, and vegetation growth during the breeding season (Gunnarsson and Tómasson 2011).

### 2.5 Statistical Analysis

To explore phenological variation within the Icelandic breeding wader community, we performed linear mixed models (LMM) with first arrival date (FAD) and laying dates (LD) as response variables and migration distance, year, and their interaction as fixed effects. Species was included as a random intercept to account for non‐independence within species. We explored regional variation in laying dates but, as we found no evidence for regional variation in our dataset, we excluded region from the analysis to simplify model structure.

We then replaced year in these LMMs with arrival temperature (at) and laying temperature (lt) to examine the influence of annual variation in spring temperature in Iceland on timing of arrival and breeding, respectively, and test whether temperature effects vary with migration distance. In all models, we used migration distance on the log_10_ scale and year centred to 2014 (year 0 = 2014).

Data were analysed in R 4.0.2 (R Core Team 2020). Packages *lme4* (Bates et al. 2015) and *lmerTest* (Kuznetsova et al. 2017) were used for the mixed models, and the package *performance* (Lüdecke et al. 2021) was used to check model's assumptions.

## 3 Results

### 3.1 Migration Distance and Timing of Arrival and Laying in Icelandic Waders

**FIGURE 2:**
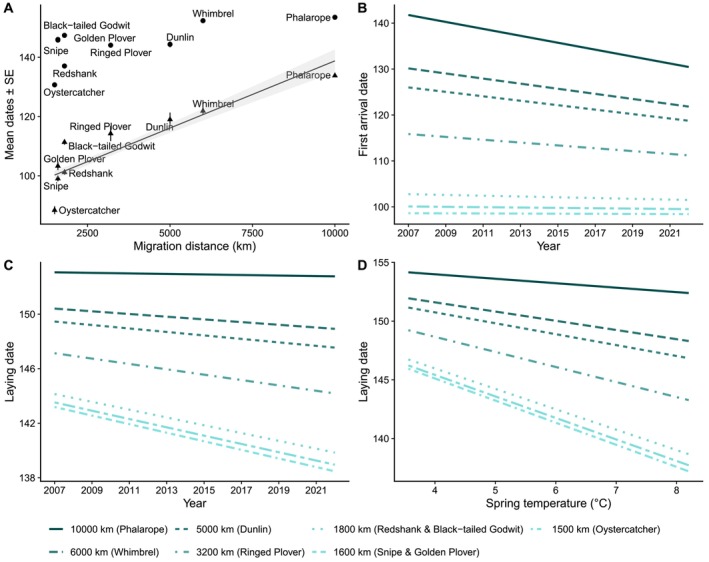
(A) Variation in Julian dates of first arrival (triangles) and laying (dots) for nine Icelandic wader species that migrate over different distances. The solid line shows the significant predicted relationship, with the shaded area indicating the standard error. Predicted annual variation in (B) first arrival and (C) laying dates in relation to migration distances. (D) Predicted relationship between laying dates and spring temperatures in Iceland across migration distances.

The nine wader species undertook migratory journeys from their breeding grounds in Iceland that ranged from short‐distance movements to W Europe (1500–1800 km) to long‐distance movements to W Africa (6000 km) or S America (10,000 km) (Figure 1). Mean first arrival dates (calculated across the study period for each species) in Iceland spanned 46 days, with Oystercatcher appearing first (in late March) in central southern Iceland and Red‐necked Phalarope last (early May; Table S1 and Figure 2). The number of recorded nesting attempts varied among species, from fewer than 100 nests for Red‐necked Phalarope to more than 1000 nests for Whimbrel and over 2500 nests for Oystercatcher (Table S1 and Figure S1). Laying dates also varied considerably among species, with Oystercatcher laying earliest on average, and Red‐necked Phalarope last (Table S1 and Figure S1). However, the range in the mean timing of laying across species was considerably smaller than the range in the timing of arrival, spanning a period of 22 days. The mean arrival‐laying gap (defined as the interval between the first arrival date and the first known laying date for each species) ranged from just over a week (Dunlin) to 25 days (Snipe) (Table S1).

### 3.2 Effects of Migration Distance on Migratory and Breeding Phenology

Species with a shorter migration distance arrived significantly earlier than species travelling longer distances, but the relationship between laying dates and migration distance was not significant, mostly due to large variation in laying dates among species travelling shorter distances (Figure 2A and Table 1).

**TABLE 1:** Results of linear mixed models exploring the variation in (A) first arrival date and (B) laying date of nine wader species over 16 years in relation to migration distance, year, and their interaction. *t*‐tests use Satterthwaite's method.

			Estimate	SE	df	*t*	*P*	Variance	SD	*R* ^2^	
										Marginal	Conditional
A.	First arrival date (*n* = 105)									0.715	0.897
	Fixed effects:										
		Intercept	–47.80	24.24	8.18	–1.97	0.083				
		Migration distance	20.01	3.06	8.37	6.55	**< 0.001**				
		Year	2.84	1.45	98.23	1.97	0.052				
		Migration distance: year	–0.39	0.19	98.42	–2.08	**0.040**				
	Random effects:										
		Species						32.21	5.68		
		Residual						18.25	4.27		
B.	Laying date (*n* = 5993)									0.096	0.271
	Fixed effects:										
		Intercept	94.95	22.52	7.28	4.22	**0.004**				
		Migration distance	6.30	2.83	7.31	2.23	0.060				
		Year	–1.45	0.56	5986.72	–2.58	**0.010**				
		Migration distance: year	0.16	0.07	5986.94	2.18	**0.030**				
	Random effects:										
		Species						30.61	5.53		
		Residual						127.56	11.29		

*Note:* Bold values indicate statistically significant effects (*p* < 0.05).

### 3.3 Effects of Migration Distance on Changes in Migratory and Breeding Phenology

Whereas laying dates of the Icelandic wader community advanced significantly over the 16 years of the study, first arrival date did not (Table 1). The interaction between migration distance and year was significant in both models but with opposite directions (Table 1); advances in the timing of arrival were greater for species travelling over longer distances for the period considered here (Figure 2B), whereas advances in the timing of laying were greater for species travelling over shorter distances (Figure 2C). Short‐distance migrants typically arrived in early April and laid during mid‐May, with lay dates advancing by almost a week during the study period. By contrast, long‐distance migrants typically arrived in early‐mid May and laid in late May/early June and, despite their arrival advancing by approximately a week during the study period, lay dates advanced by a couple of days (Figure 2C). It should be noted that in the laying dates model (Table 1B), the marginal *R* ^2^ (i.e., variation explained by the fixed factors) was low compared to the conditional *R* ^2^ (i.e., variation explained by the fixed and random effects), suggesting that migration distance and year explained less of the variance in laying dates than the variation within and across species (random effect).

### 3.4 Effects of Spring Temperature on Migratory and Breeding Phenology

Spring temperatures in Iceland during the arrival period were not associated with the timing of arrival, and this did not vary for species migrating over different distances (Table 2A). However, laying dates were earlier in warmer years, and species migrating shorter distances showed greater advances in lay dates in warmer springs than species migrating further (significant positive interaction between migration distance and temperature, Table 2B and Figure 2D). The marginal *R* ^2^ was again low compared to the conditional *R* ^2^ (Table 2B), suggesting that migration distance and spring temperature explained less of the variance in laying dates than the variation within and across species.

**TABLE 2:** Results of linear mixed models examining the effect of migration distance, spring temperature, and their interaction on the timing of (A) arrival and (B) laying of nine wader species over 16. Spring temperature was calculated as the mean of March and April for the timing of arrival (at) and April to June for the timing of laying (lt). T‐tests use Satterthwaite's method.

			Estimate	SE	df	*t*	*P*	Variance	SD	*R* ^2^	
										Marginal	Conditional
A.	First arrival date (*n* = 105)									0.708	0.892
	Fixed effects:										
		Intercept	–8.61	29.04	18.18	–0.30	0.770				
		Migration distance	15.25	3.70	19.19	4.13	**0.001**				
		Arrival temperature (at)	–11.91	7.78	95.13	–1.53	0.129				
		Migration distance: at	1.36	1.00	95.21	1.35	0.180				
	Random effects:										
		Species						30.49	5.52		
		Residual						18.04	4.25		
B.	Laying date (*n* = 5993)									0.093	0.295
	Fixed effects:										
		Intercept	141.62	28.14	12.90	5.03	**< 0.001**				
		Migration distance	1.51	3.57	13.48	0.42	0.679				
		Laying temperature (lt)	–7.68	2.10	5986.42	–3.66	**< 0.001**				
		Migration distance: lt	0.79	0.27	5986.74	2.90	**0.004**				
	Random effects:										
		Species						35.98	6.00		
		Residual						125.46	11.20		

*Note:* Bold values indicate statistically significant effects (*p* < 0.05).

## 4 Discussion

Shifts in the timing of key events in the annual cycle of organisms are occurring across the globe in response to rapid environmental changes (Garcia et al. 2014; Parmesan and Yohe 2003; Walther et al. 2002). These phenological shifts have the potential to directly influence individual fitness and drive population‐scale changes in abundance and distribution (Alves et al. 2019). However, the magnitude of phenological change can vary greatly among species and, for migratory species, is often greater for those travelling over shorter distances. Here, we demonstrate that the timings of spring arrival and laying in Icelandic breeding waders vary with migration distance, with species travelling from closer wintering grounds arriving and laying earlier than species travelling further. While laying dates have advanced across all species, the advances are greater for short‐distance migrants, which typically arrive more than a month before laying. In contrast, long‐distance migrants, which arrive considerably later in the season, have shown much smaller advances in laying, even though their arrival dates have advanced slightly. Laying dates are also earlier in warmer springs, particularly for species travelling shorter distances. These results suggest that opportunities to advance nesting in warmer springs are much greater for short‐distance migrants that arrive in March/April and can lay from mid to late May, depending on conditions, than for long‐distance migrants that do not arrive until May and breed soon after arrival.

### 4.1 Migration Distance and Phenology

Migration distance plays an important role in migratory phenology, with long‐distant migrants usually arriving later at the breeding grounds than short‐distance migrants (Alves et al. 2013b; English et al. 2025). However, our results show that the effect of migration distance on timing of laying is less clear, largely because laying dates are strongly influenced by local environmental conditions (Charmantier and Gienapp 2014; Lameris et al. 2018; Shave et al. 2019). Among species travelling shorter distances, which generally arrive earlier than those travelling further, variation in timing of laying can be substantial (Figure 2A). This suggests that laying is to some extent independent of timing of arrival and more responsive to post‐arrival environmental conditions (Alves et al. 2019; English et al. 2025; Laidlaw et al. 2020; Lourenço et al. 2011; Low et al. 2019; van den Brink et al. 2008), as well as the arrival of their mates, given that most of these species show high levels of mate fidelity (Gunnarsson et al. 2004; Handel and Gill 2000). For example, Icelandic Black‐tailed Godwits and Golden Plovers have a relatively long period between arrival and laying compared to other species that travel similar distances (Figure 2A). In godwits, this interval likely reflects the need to conceal nests and wait for sufficient vegetation growth, particularly in cold springs (Alves et al. 2019; Laidlaw et al. 2020), whereas Golden Plovers breed across a wide altitudinal gradient (Méndez et al. 2025), which is likely to result in considerable variation in lay dates. Thus, the early arrival of species wintering closer to their breeding grounds does not necessarily guarantee early laying, as the timing of laying is ultimately more strongly influenced by local environmental conditions than by migration distance or spring arrival. For long‐distant migrants, the later arrival is likely to mean that conditions for breeding are suitable upon arrival and, thus, timing of breeding is likely to be more influenced by the timing of arrival (Low et al. 2019).

### 4.2 Changes in Migratory and Breeding Phenology

The timing of arrival of many migratory species in Iceland advanced markedly (by approximately 2 weeks) during the 1990s and 2000s, when spring temperatures were increasing rapidly (Gunnarsson and Tómasson 2011). However, in the present study (2007–2022), a period of relatively stable spring temperatures (Figure S2), advances in arrival were observed only among species travelling long distances and showed no association with spring temperatures in Iceland. The recent advances (of almost 1 week) in spring arrival among long‐distance migrants could be linked to processes occurring at their non‐breeding grounds (Lameris et al. 2018, 2025; Rakhimberdiev et al. 2018) or could reflect generational increases in the frequency of early‐arriving individuals in these populations, as has been shown for shorter‐distance species (Gill et al. 2014). While our measure of first arrival date at the inland site could be affected by unusually early individuals, such measures correlate well with the main arrival wave of these species (Gill et al. 2014; Gunnarsson et al. 2006), suggesting that our estimates reliably capture relative changes in migratory timing.

The differing patterns of change in migration and breeding phenology among species migrating over different distances highlight the importance of the interval between arrival and laying. In this study, species overwintering closer to Iceland typically arrived 4–6 weeks before long‐distance species and nested 4–5 weeks after arrival (Figure 2A) and appear to use this interval to initiate laying early in warmer springs. The advances in lay dates of these species, and their earlier laying in warmer springs, suggest that they are taking advantage of an increasing frequency of years with conditions suitable for early laying. By contrast, species travelling longer distances typically nested within 2–3 weeks of arrival. Their later arrival likely means that conditions are already suitable for nesting in most years upon their arrival, resulting in smaller advances in laying than those seen in short‐distance species.

Our results are consistent with previous work showing that the period between arrival and laying provides an opportunity to respond to annual variation in local conditions (Gill et al. 2014; Low et al. 2019; Nicolau et al. 2021; Smith and Fraser 2024), particularly for individuals or species travelling shorter distances (Low et al. 2019). Although we could not test explicitly for temporal trends in arrival‐laying intervals due to the nature of our data (one first arrival date per species per year), the finding that short‐distance migrants in the Icelandic wader community did not advance arrival but did advance laying during this period suggests that this interval has become shorter. By contrast, the fact that species migrating longer distances slightly advanced both arrival and laying during this period suggests that their arrival‐laying interval has remained relatively constant, likely reflecting the minimum time needed for pre‐laying activities such as recovering from migration, finding a mate, and locating a suitable nest site. These contrasting responses highlight how migration distance, through its influence on the timing of arrival, can influence the capacity of species to adjust breeding schedules to changing spring conditions.

Many long‐distance migratory species are currently declining, in contrast to short‐distance migrants (Vickery et al. 2023; Zurell et al. 2018). Given the benefits associated with early nesting, including greater offspring recruitment rates (Alves et al. 2019; Lok et al. 2017; Morrison et al. 2019), the advancing timing of laying in shorter‐distance species could be contributing to these divergent population trajectories. Much of the focus on migratory species' responses to climate change has been on the potential for advances in lay dates to reduce mismatches in the timing of peak food abundance for chicks (Both et al. 2010; Huchler et al. 2020; Reneerkens et al. 2016; Renner and Zohner 2018). However, opportunities to quantify variation in migratory and breeding phenology across species remain rare, and our findings highlight the potential for earlier arrival of short‐distance migrants to provide greater opportunities to advance laying, and thus to accrue the benefits of breeding early (Méndez et al. 2022; Morrison et al. 2019). By contrast, the constraints imposed by later arrival on longer‐distance migrants could limit their opportunities to accrue these benefits. While most existing work has focused on investigating links between migratory strategy and breeding phenology or reproductive success within populations (e.g., Alves et al. 2012; Grist et al. 2017; Kentie et al. 2017, 2023; Méndez et al. 2022), comparative studies across species differing in migration distance while experiencing similar local breeding conditions remain scarce. Such studies would help to identify the costs and benefits of migration distance and timings of spring arrival during this period of rapid environmental change at high latitudes.

## 5 Conclusions

Across a community of breeding waders that travel substantially different distances to their non‐breeding grounds, we show that species travelling shorter distances arrive on the breeding grounds earlier and are advancing lay dates more than species travelling longer distances. Short‐distance migrants nest much earlier in warmer springs than longer‐distance species, suggesting that their early arrival allows them to take advantage in years when conditions are suitable for earlier nesting. These findings suggest that the later arrival of long‐distance migrants limits their opportunities to respond to warming springs. Given the benefits of nesting early, the constraint of arriving too late to take advantage of early springs during this period of rapid environmental change could be contributing to the divergent population trajectories of many short‐ and long‐distance migratory species.

## Author Contributions

**Verónica Méndez:** conceptualization (equal), data curation (lead), formal analysis (lead), funding acquisition (equal), investigation (lead), methodology (lead), resources (equal), supervision (equal), validation (equal), visualization (lead), writing – original draft (lead), writing – review and editing (lead). **José A. Alves:** conceptualization (equal), funding acquisition (equal), investigation (supporting), resources (equal), supervision (equal), writing – review and editing (supporting). **Jennifer A. Gill:** conceptualization (equal), funding acquisition (equal), investigation (supporting), resources (equal), supervision (equal), writing – review and editing (supporting). **Böðvar Þórisson:** conceptualization (supporting), data curation (supporting), resources (equal), writing – review and editing (supporting). **Camilo Carneiro:** conceptualization (supporting), resources (equal), writing – review and editing (supporting). **Aldís E. Pálsdóttir:** resources (supporting), writing – review and editing (supporting). **Sölvi R. Vignisson:** resources (supporting), writing – review and editing (supporting). **Gunnar Tómasson:** resources (supporting), writing – review and editing (supporting). **Tómas G. Gunnarsson:** conceptualization (equal), funding acquisition (equal), investigation (supporting), methodology (supporting), resources (equal), supervision (supporting), writing – review and editing (supporting).

## Funding

This work was supported by the Icelandic Research Fund (217587‐051, 228625‐051 and 217753‐051), University of Iceland Research Fund, Natural Environment Research Council, NE/M012549/1, National Funds through FCT—Fundação para a Ciência e a Tecnologia I.P., under the project CESAM‐Centro de Estudos do Ambiente e do Mar, references UID/50017/2025 (https://doi.org/10.54499/UID/50017/2025) and LA/P/0094/2020 (https://doi.org/10.54499/LA/P/0094/2020), and ProPolar.

## Conflicts of Interest

The authors declare no conflicts of interest.

## Supporting information


**Appendix S1:** ece373080‐sup‐0001‐AppendixS1.docx.

## Data Availability

Data are available from the Dryad Digital Repository https://doi.org/10.5061/dryad.qz612jmtn (Méndez et al. 2025).
