# Supplementary material for: Effects of Migration Distance on Shifting Migratory and Breeding Phenology in Waders
Source: Ecol Evol. 2026 Feb 27;16(3):e73080. doi: 10.1002/ece3.73080 (PMC12949201; doi:10.1002/ece3.73080)
Supplement: Supplementary file 1 — Appendix S1: ece373080‐sup‐0001‐AppendixS1.docx. [file ECE3-16-e73080-s001.docx]

**Supporting information**

**Table S1**. Clutch size, incubation length and laying intervals used to estimate laying dates, along with sample sizes of nests included in the analysis. Numbers in brackets indicate the number of excluded ‘late’ nests. Nests excluded from the analysis were either known renesting attempts or classified as ‘late’ nests for each species and year (defined using Tukey’s boxplot rule: >1.5 times the interquartile range of laying dates above the 75^th^ percentile). Mean first arrival date (FAD; days since 1 Jan), mean laying date and mean gap between arrival and laying (arrival date – first known date of nest initiation) are presented as averages across the study period (2007-2022).

| **Species** | **Clutch size** | **Incubation length (days)** | **Laying interval (days)** | **Total nests (‘late’ nests)** | **Known renesting attempts** | **Mean FAD (SE)** | **Mean laying date (SE)** | **Mean arrival-laying gap (SE)** |
| --- | --- | --- | --- | --- | --- | --- | --- | --- |
| Oystercatcher^1^ | 3 | 26 | 1 | 2609 (44) | 166 | 88 (1.3) | 131 (0.2) | 22 (1.1) |
| Snipe^1^ | 4 | 19 | 1 | 343 (5) | 0 | 99 (0.9) | 146 (0.7) | 25 (1.2) |
| Golden Plover^1^ | 4 | 29 | 1.25 | 386 (7) | 0 | 103 (1.3) | 146 (0.8) | 21(1.4) |
| Black-tailed Godwit^1^ | 4 | 23 | 1.25 | 239 (4) | 1 | 111 (0.8) | 147 (0.6) | 22 (1.5) |
| Redshank^1^ | 4 | 24 | 1.25 | 316 (6) | 0 | 101 (0.8) | 137 (0.6) | 19 (1.6) |
| Ringed Plover^1^ | 4 | 25 | 1.25 | 764 (13) | 99 | 114 (2.5) | 144 (0.4) | 13 (3.9) |
| Dunlin^1^ | 4 | 22 | 1.25 | 119 (3) | 0 | 119 (2.3) | 144 (1) | 8 (3.4) |
| Whimbrel^2^ | 4 | 26 | 0.85 | 1132 (18) | 19 | 122 (1.2) | 152 (0.3) | 15 (1.5) |
| Red-necked Phalarope^3^ | 4 | 21 | 1 | 85 (3) | 0 | 134 (0.6) | 153 (0.8) | 13 (2.1) |

*^1^ Cramp & Simmons (1983)*

*^2^ Grant (1989)*

*^3^ Brown (2010)*

**Table S2**. Total of nests with estimated laying date for each species and region. Note that given the small sample size in the East (E) and Southeast (SE) of Iceland, we did not include data from these regions in the analysis.

| **Species** | **S** | **SW** | **W** | **NW** | **NE** | **E** | **SE** | **Total** |
| --- | --- | --- | --- | --- | --- | --- | --- | --- |
| Oystercatcher | 945 | 684 | 68 | 788 | 124 | 108 | 6 | **2889** |
| Snipe | 311 | 10 | 14 | 5 | 3 | 0 | 2 | **345** |
| Golden Plover | 326 | 17 | 14 | 27 | 2 | 0 | 5 | **391** |
| Black-tailed Godwit | 234 | 2 | 0 | 3 | 0 | 0 | 0 | **240** |
| Redshank | 224 | 22 | 21 | 49 | 0 | 2 | 0 | **318** |
| Ringed Plover | 186 | 42 | 22 | 512 | 2 | 3 | 1 | **867** |
| Dunlin | 104 | 4 | 4 | 1 | 6 | 0 | 1 | **120** |
| Whimbrel | 1041 | 28 | 19 | 43 | 1 | 2 | 0 | **1153** |
| Red-necked Phalarope | 84 | 0 | 0 | 0 | 1 | 0 | 0 | **85** |
| **Total** | **3546** | **865** | **162** | **1558** | **147** | **115** | **15** | **6408** |

**Table S3**. Results of linear mixed models of the variation in laying date when excluding ‘late’ nests of nine wader species over 16 years in relation to migration distance and year. T-tests use Satterthwaite’s method.

**Figure S1**. Distribution of laying dates across the nine wader species included in this study.

*
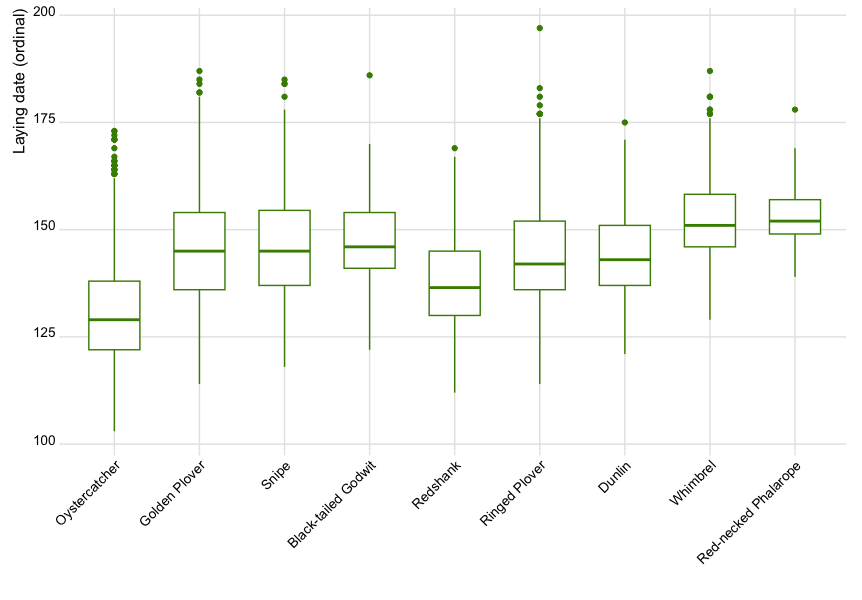
*

**Figure S2**. Annual variation in the mean monthly temperature ± se (A-C) and the mean for the entire breeding season (D) across the five regions included in this study.

*
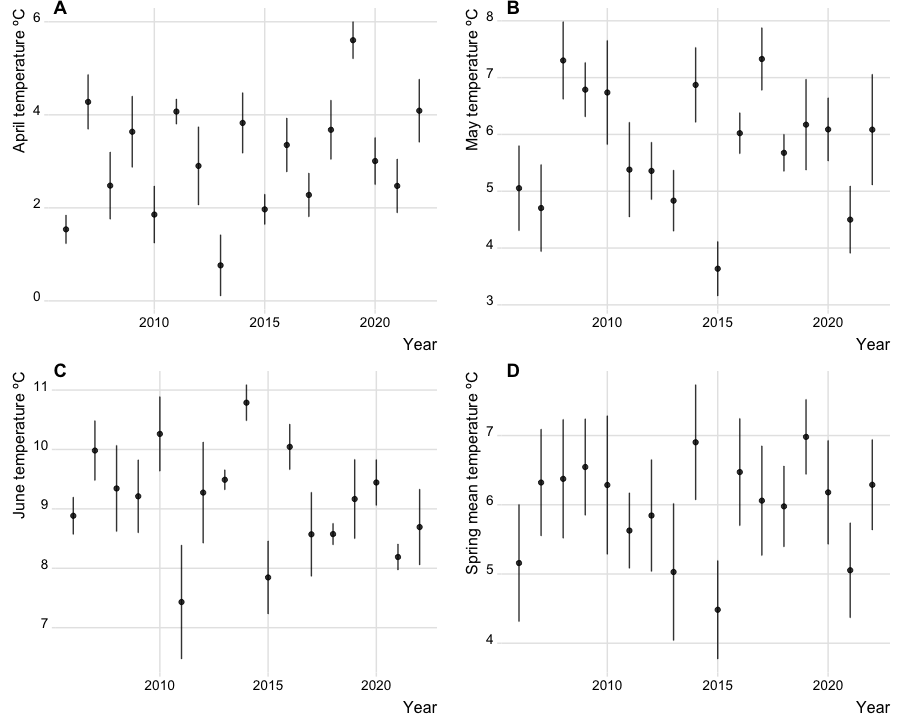
*
